# Effect of empathic ability on white lie cognition among autism spectrum disorder and intellectual disabilities

**DOI:** 10.3389/fpsyg.2026.1775180

**Published:** 2026-05-19

**Authors:** Yunjie Liao, Wenjing Fu, Zhen Zeng, Fan Liu, Jun Peng

**Affiliations:** 1 School of Education Science, Hunan Normal University, Changsha, Hunan, China; 2 Department of Radiology, The Third Xiangya Hospital, Central South University, Changsha, Hunan, China; 3 Yucai Dongtun Primary School, Changsha, Hunan, China; 4 School of Education Science, Huaihua University, Huaihua, Hunan, China; 5 Xiangtan Institute of Technology, Xiangtan, Hunan, China; 6 Clinical and Technical Support, Philips Healthcare, Guangzhou, Guangdong, China

**Keywords:** autism spectrum disorder, children with intellectual disabilities, cognitive empathy, emotional empathy, white lie cognition

## Abstract

In interpersonal communication, white lies are often used to consider the feelings of others or to avoid the negative consequences of telling the truth. Understanding the relationship between children’s empathic ability and white lie cognition cannot only help to comprehend the development process of children’s white lies and deepen the research in children’s moral development and social cognition, but also provide a theoretical basis for educating children on white lie cognition and fostering prosocial behavior. Although white lie cognition is well-documented in children with typical development, the role of empathic ability in this process remains under-researched in neurodiverse populations. This study examined the influence of empathic ability on white lie cognition in children with autism spectrum disorder and children with intellectual disability. A white lie cognition task and an empathy continuum test were administered to assess the performance and differences among children with typical development, ASD, and ID across various dimensions of white lie cognition and empathic ability. Results: (1) Significant differences in cognitive empathy, emotional empathy, and evaluations of both the degree and morality of white lies were observed among autism spectrum disorder, children with intellectual disabilities, and children with typical development. The stronger a child’s cognitive and emotional empathy, the easier it was to evaluate the degree of white lie and its moral implications. (2) Autism spectrum disorder demonstrated significantly lower empathic ability and white lie cognition compared with those with intellectual disabilities. Moreover, greater empathic ability in these children was associated with improved white lie cognition. (3) Across children with typical development, autism spectrum disorder, and children with intellectual disabilities, when empathic ability scores were higher, cognitive empathy and emotional empathy could positively predict all aspects of white lie cognition. These results indicate that the empathic ability of autism spectrum disorder and children with intellectual disabilities can directly affect their white lie cognition. In these children, higher empathic ability is associated with improved cognition of white lies and promotes accurate evaluations of both the degree and morality of white lies.

## 1 Introduction

A white lie, often described as a well-intentioned falsehood, refers to a statement that deviates from the truth to benefit the listener. It is told with the understanding that honesty might provoke a negative response while telling a lie would result in a positive reaction (Heyman et al., 2020; Gallant et al., 2020). Psychologists have long been concerned with children’s cognition of white lies. Piaget noted that white lies are prevalent in everyday life, combining elements of deception and altruism, and play a crucial role in establishing, maintaining, and promoting interpersonal relationships (Terkourafi, 2023). Subsequently, psychologists have studied how children learn to tell white lies and how morality and reasoning contribute to their understanding of such behavior (Cohen and Strayer, 1996). In recent years, white lie cognition has gained attention in the research on children’s moral development and social cognition (Demedardi et al., 2021).

White lie cognition refers to an individual’s capacity to understand, interpret, evaluate, and reason regarding white lies (Demedardi et al., 2021). It mainly includes 3 aspects: conceptual understanding, degree evaluation, and moral evaluation (Bussey, 1999). Conceptual understanding refers to the individual’s ability to accurately infer the mental state of a liar who lies to protect another’s feelings and preserve positive emotions. Degree evaluation refers to the individual’s ability to precisely judge the extent to which a lie is motivated for the benefit of others. Moral evaluation of white lies refers to the process by which individuals use existing moral concepts to judge and assess the right/wrong, good/bad, and virtuous/vicious nature of lying for the benefit of others. Research indicates that children with typical development (TD) are able to express judgments regarding white lies as early as age 3 (Schlenker and Britt, 2001) and begin to understand white lies from the perspective of lying by age 4 (Kokina and Kern, 2010). In contrast, autism spectrum disorder demonstrate a delay of approximately 7 years in the acquisition and development of white lies compared with those with TD (Williams White et al., 2007), whereas children with intellectual disability (ID) lag by approximately 6 years (Lin et al., 2023). A few studies indicate that because children with ASD and ID acquire a conceptual understanding of white lies later than children with TD, their moral evaluation of white lies also emerges at a later age (Williams White et al., 2007; Lin et al., 2023). Additionally, as children with TD grow older, their assessment of the severity of a white lie gradually shifts from viewing it as “a very serious big lie” to regarding it as “an acceptable small lie” (Talwar and Crossman, 2011).

Research indicates that children with TD are able to grasp the concept of white lies by age 4, and this understanding continues to improve with age (Bussey, 1999). Other studies suggest that moral evaluations of white lies among children with TD aged 3–5 years become increasingly negative as they grow older; however, these evaluations remain more positive than those directed toward harmful lies (Talwar et al., 2007). Children also view white lies as more acceptable than harmful lies (Zhen and Liming, 2024). Popliger et al. (2011) observed that higher levels of moral approval of white lies in children with TD are associated with a greater tendency to use white lies when attempting to cover up their own mistakes (Popliger et al., 2011; Lim et al., 2020). For autism spectrum disorder, their moral evaluation in white lie cognition is more likely to rely on direct factual statements due to their delayed acquisition and development of white lies (Williams White et al., 2007; Li et al., 2011; Baron-Cohen, 1992). Williams White et al. (2007) observed that the level of moral evaluation of white lies in children with ASD affects the development of their social interaction, interpersonal relationships, and white lie cognition. For children with ID, the proportion of those who express false responses increases with age; older children demonstrate greater ability to understand the mental state of a gift-giver (who may need to describe the gift verbally, which might result in an awkward feeling embarrassed) (Lin et al., 2023). These findings indicate that children with TD have developed a certain level of conceptual understanding and moral evaluation of white lies, but research on white lie cognition in children with ASD and ID remains insufficient. With the advancement of special education in China, researchers have begun to place increasing emphasis on the development of white lie cognition in children with ASD and ID (Talwar and Crossman, 2011; Ma et al., 2011). White lies are motivated by a desire to protect others’ feelings. When forming cognitive judgments regarding the nature of white lies, individuals must interpret the mental states of both the speaker and the listener. Consequently, the ability to accurately perceive and understand others’ emotional states is particularly crucial (Evans and Lee, 2013). This ability to generate emotional experiences that correspond to others’ emotions and feelings, and to regulate empathy at the cognitive level while influencing the intensity of empathic experience, is referred to as empathic ability (Zhang et al., 2024; Buchanan et al., 1991). According to Piaget’s theory of moral cognitive development, moral evaluation emerges from the coordination of cognitive processing and emotional processing (Helion and Ochsner, 2018). Research has found that children with typical development are able to make judgments about white lies as early as age 3 (Schlenker and Britt, 2001) and begin to understand white lies from the perspective of lying by age 4 (Kokina and Kern, 2010). As they grow older, their evaluation of the severity of white lies gradually shifts from viewing them as “very serious big lies” to regarding them as “acceptable small lies” (Talwar and Crossman, 2011). Furthermore, although moral evaluations of white lies among children with typical development aged 3–5 years become increasingly negative with age, these evaluations remain more positive than those directed toward harmful lies (Talwar et al., 2007; Zhen and Liming, 2024). Huebner et al. (2009) observed that children mainly rely on behavioral outcomes as the primary basis for moral evaluation; however, empathy and emotional factors also contribute by moderating the influence of factual information on moral evaluation of white lies. Empathy includes not only an individual’s understanding of others’ emotional states (i.e., cognitive empathy) but also the emotional experiences generated by the individual after experiencing others’ feelings in a situation (i.e., emotional empathy) (Dvash and Shamay-Tsoory, 2014). The two components follow different developmental trajectories: cognitive empathy emerges earlier, while emotional empathy develops gradually during early childhood. As children mature, these two components become increasingly integrated (Dvash and Shamay-Tsoory, 2014; Decety et al., 2018). For white lie cognition to develop, the liar must first recognize that telling the truth would elicit a negative emotional response from the listener, while telling a lie would produce a more positive emotional reaction, and must also be able to adopt the listener’s perspective (Barr and Higgins-D’Alessandro, 2007; Mesurado and Richaud, 2017). Thus, empathic ability is likely to play a crucial role in white lie cognition. A few scholars have emphasized that children with ASD exhibit deficits in developing emotional empathy and reduced abilities to perceive and imitate emotional expressions, which, in turn, contribute to delays in the development of white lie cognition (Michael et al., 2007). For children with ID, researchers have assessed empathic ability using scales and observed it to be a medium or medium-low level, with unbalanced internal development–emotional empathy tends to be more developed than cognitive empathy (Lin et al., 2023). However, the influence of empathic ability on white lie cognition in children with ID has not been systematically explored. In summary, empathy has been demonstrated to facilitate prosocial behaviors such as sharing and helping behaviors (Ozdemir, 2008; Klimecki et al., 2016). Children telling white lies is a complex prosocial behavior, yet previous studies have not explored whether white lie cognition in children with ASD and ID is influenced by empathic ability.

White lies represent a crucial component of children’s social behavior. However, laboratory investigation of white lies remains challenging due to the complexity of their use and interpretation, and the underlying cognitive processes are not yet fully understood. Children with ASD and ID constitute a substantial proportion of children with special needs, rendering it particularly crucial to examine their understanding of white lies and the factors that may influence this process. Existing research has largely focused on white lie cognition in children with TD, with limited attention to its development in children with ASD and ID, and even less focused on the potential role of empathic ability across various components of white lie cognition. A clearer understanding of these relationships may provide a theoretical basis for supporting the development of prosocial behavior and social understanding in these populations. To address these shortcomings of previous research, this study aimed to examine white lie cognition in children with ASD and children with ID, and explore whether empathic ability plays a role in various aspects of white lie cognition.

Based on the aforementioned theoretical considerations and empirical findings, the following hypotheses were proposed. Research Hypothesis 1: Compared with children with TD, children with ASD and ID demonstrate marked differences in the development of white lie cognition. Research Hypothesis 2: Compared with low empathic ability, children with ASD and ID exhibit more mature white lie cognition under conditions of high empathic ability. Furthermore, this enables targeted intervention design for children with autism spectrum disorder and children with intellectual disability. If emotional empathy proves to be a stronger predictor, interventions should focus on emotion recognition and sharing; if cognitive empathy is more critical, then perspective-taking training should be emphasized.

## 2 Materials and methods

### 2.1 Participants

The required sample size was determined based on Faul et al. (2007). Using an effect size of *f* = 0.3, an α = 0.05, and a power of 1 β = 0.95, the minimum total sample size needed for this study was calculated to be 145. A total of 150 participants were actually recruited and were categorized into three groups of various age ranges: children with TD, children with ASD, and children with ID.

Fifty children with TD (26 boys) were selected from a kindergarten in Hunan Province, and 50 children with ASD (24 boys) were matched to the children with TD in terms of chronological age and mental age. All participants were clinically diagnosed with autism spectrum disorder (ASD) by qualified healthcare professionals and possess formal diagnostic documentation issued by accredited medical institutions. Comorbid developmental disorders were systematically excluded through comprehensive clinical assessment, and all participants fully satisfy the diagnostic criteria for ASD outlined in the fifth edition of the Diagnostic and Statistical Manual of Mental Disorders (DSM-5). Additionally, 50 children with ID (23 boys) were also recruited; these children were classified as having moderate intellectual disability, with intelligence quotient scores between 30 and 50, as reported by their schools. The children with ASD and ID were recruited from a special education school and an inclusive school in Hunan Province.

Informed consent was obtained from the parents or guardians of all participants, and the study was reviewed and approved by the institutional ethics committee.

Prior to the experiment, participants underwent following assessments: Children with ASD were evaluated using CARS, and all participants completed the Raven’s Progressive Matrices and the Peabody Picture Vocabulary Test to measure reasoning intelligence and verbal intelligence, respectively, for all participants.

### 2.2 Experimental design

A mixed experimental design was employed, featuring 3 groups (children with TD/children with ASD/children with ID) × 2 levels of empathic ability (Cognitive empathy/emotional empathy). The dependent variable was the behavioral experimental result (the ability of white lie cognition).

### 2.3 Measures

#### 2.3.1 Reasoning intelligence test

The Standard Raven’s Progressive Matrices, compiled by J. C. Raven and revised by Qiu et al. (2020), was used to measure the participants’ intelligence level. Following the test’s scoring guidelines, raw scores were converted into percentile ranks, and participants with percentile ranks < 25% were excluded from the study. The percentile-based exclusion criterion was originally designed based on the norm-referenced framework for children with typical development. If strictly applied to children with intellectual disability, it would severely compromise the sample representativeness of this population. Therefore, this exclusion criterion was not applied to children with autism spectrum disorder or children with intellectual disability in the present study. Typically developing children were also not excluded based on this criterion, and all three groups were fully included in the analysis.

#### 2.3.2 Verbal intelligence test

The Chinese revised version of the Peabody Picture Vocabulary Test (Dunn and Dunn, 1981; Dunn et al., 1988) was administered. During the test, children were presented with 4 pictures and the experimenter read a word aloud, and they were asked to select the picture that corresponded to the word. Each correct response was scored 1 point. The test was discontinued if a child committed 6 consecutive errors within a set of 8 trials. The total number of correct responses (raw score) was used for statistical analysis.

#### 2.3.3 White lie cognition test

In this test, 4 scenario-based stories from Bussey (1999) were selected, which included 2 white lie stories and 2 blunt truth stories. Each story was paired with corresponding pictures to assess whether the child could distinguish between truth and lies. Children who successfully completed this discrimination phase proceeded to the white lie cognition assessment, which included 2 tasks: evaluation of the degree of white lie and its moral implications.

First, the moral evaluation scale was introduced. The experimenter engaged in brief communication and play with the child to establish rapport. Before the formal tasks, the experimenter presented cards depicting the moral evaluation scale to the child. The child was told that a red star (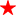) meant “good,” a black cross ( × ) meant “bad,” and a circle (○) meant “neither good nor bad.” The use of the pictorial cards ensured that all participants could understand the scale.

Second, the child’s white lie degree and moral evaluation were assessed. Stories were narrated to the child with the aid of pictures.

**FIGURE 1:**
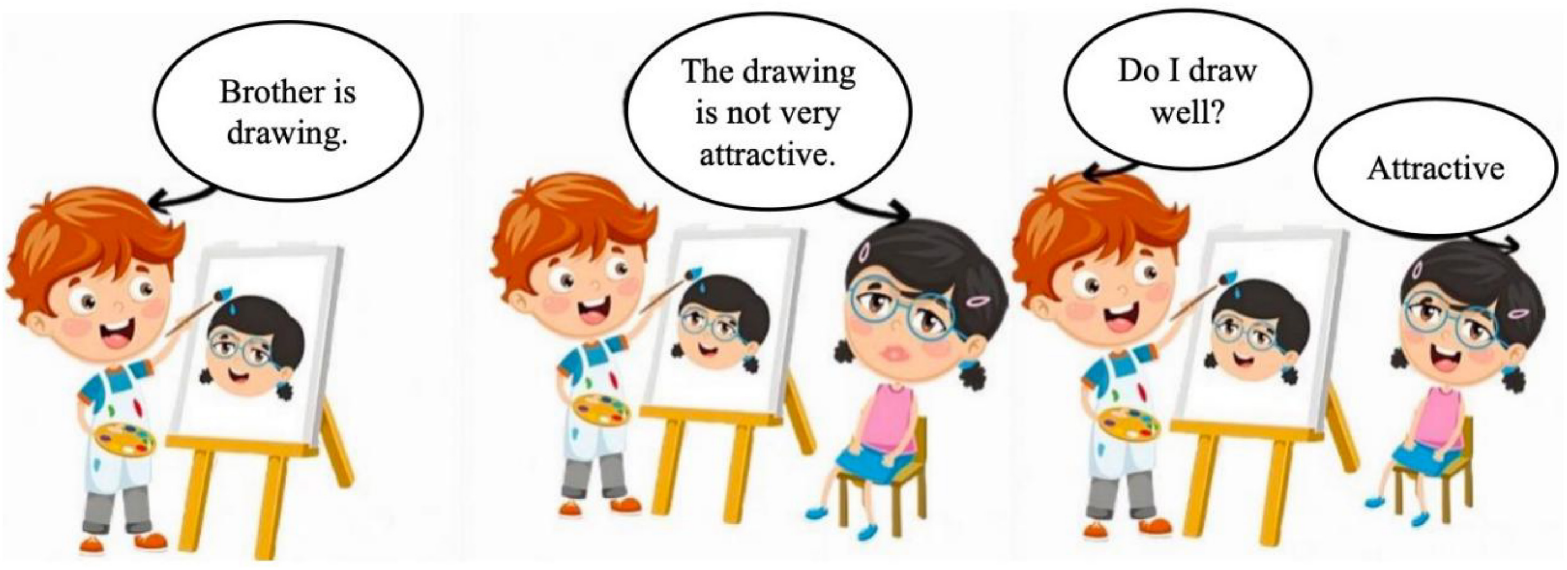
Content of the white lie stories.

Story 1 (Blunt Truth): Xiaoming’s mother baked a cake for him. Xiaoming didn’t think it tasted good. When his mother asked, Xiaoming said, “The cake doesn’t taste good!”
Story 2 (White Lie): Xiaohong’s father bought a new hat. Xiaohong didn’t think it looked nice. However, when her father asked, Xiaohong said, “The hat looks really nice!”
Story 3 (Blunt Truth): Xiaodong’s sister had a new haircut. Xiaodong didn’t think it looked nice. When his sister asked, Xiaodong said, “The haircut doesn’t look nice!”
Story 4 (White Lie): Xiaomei’s brother was drawing a picture. Xiaomei didn’t think it looked nice. However, when her brother asked, Xiaomei said, “The picture looks really nice!” (Figure 1).

After each story, the child was asked the following questions:

1. “Do you think what he/she said was the truth or a lie?” If the child answered “a lie,” a follow-up question was asked: “Was it a very big lie, a very small lie, or a medium-sized lie?”
2. “Do you think what he/she did was good, bad, or neither good nor bad? Why?” (The “why” question was to understand the reason for the moral evaluation for subsequent discussion).

After a blunt truth story, only question 1 was asked (“Do you think what the protagonist said was the truth or a lie?”), and no further questions followed. After a white lie story, question 1 was asked. If the child answered “the truth,” the assessment for that story was stopped. If the child answered “a lie,” the white lie cognition assessment (follow-up to question 1 and question 2) proceeded.

Scoring criteria:

White Lie Degree Evaluation: Lies were categorized into 3 levels: a very small lie, a medium-sized lie, and a very big lie, scored as 1, 0, and − 1 point, respectively. The scores from Story 2 and Story 4 were summed to form the total score for white lie degree evaluation.

White Lie Moral Evaluation: After the degree evaluation, the child evaluated the protagonist’s behavior. Responses of “bad,” “neither good nor bad,” and “good” were scored − 1, 0, and 1 point, respectively. The scores from the 2 white lie stories were summed to form the total score for white lie moral evaluation.

#### 2.3.4 Empathic ability task

The empathy continuum test (Talwar and Crossman, 2011) was administered. Stories depicting four emotions—happiness, sadness, anger, and fear—were narrated to the children sequentially. After each story, participants were asked to identify the protagonist’s emotional state (cognitive empathy) and report their own emotional experience (emotional empathy). Each correct response was awarded 1 point. A combined scoring method was adopted in this study: for each story, 2 points were given if both cognitive empathy and emotional empathy were correctly identified, 1 point if only one was correct, and 0 points if both were incorrect. Scores were summed across the four stories, resulting in a total score range of 0–8 for both cognitive empathy and emotional empathy. The overall empathic ability score was the sum of these two components, ranging from 0 to 16 points. The protagonist’s sex was matched to the child’s sex, and the story order was randomized.

### 2.4 Measures

**FIGURE 2:**
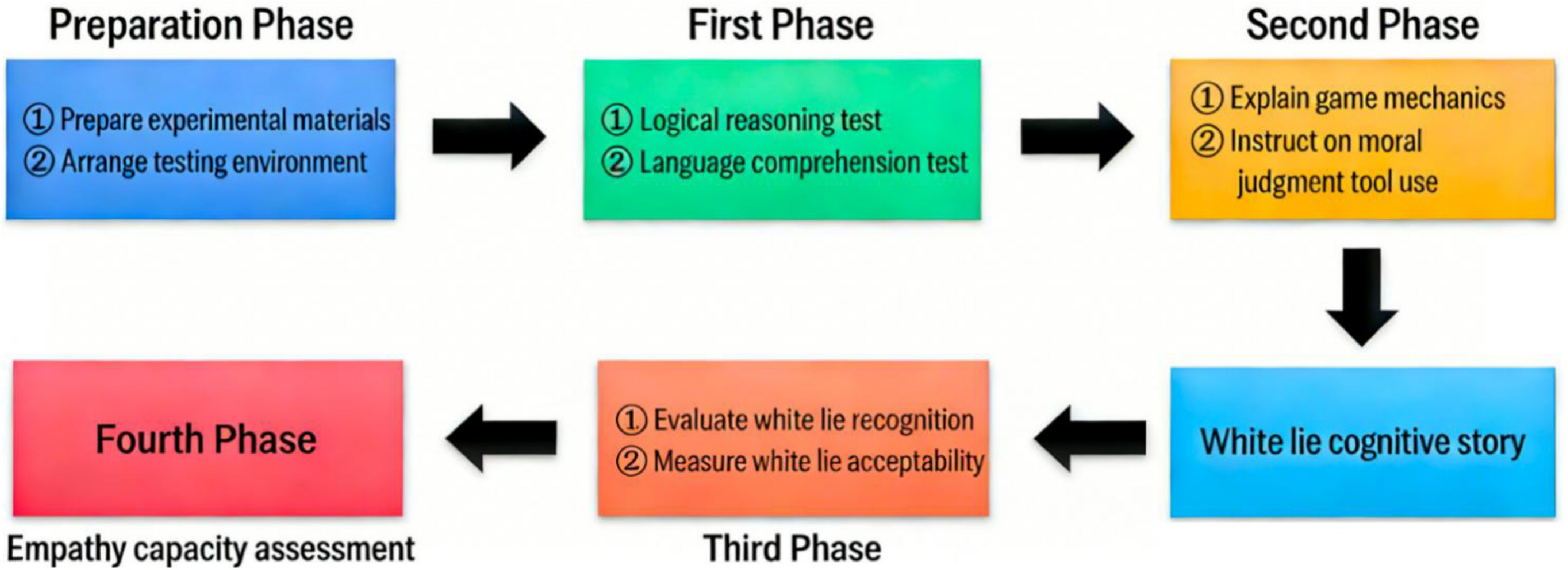
The overall experimental procedure is illustrated.

The overall experimental procedure is illustrated (Figure 2).

### 2.5 Data analysis

The collected data were entered and statistically analyzed using SPSS 24.0. Independent samples *t*-tests were conducted to analyze participants’ scores on white lie cognition and empathic ability. Correlation analyses and regression analyses were performed to examine the relationships between white lie cognition and empathic ability.

## 3 Results

### 3.1 Descriptive statistics

Since the chronological age of children with ASD and those with ID was greater than that of children with TD. The descriptive statistics of participants’ age, reasoning intelligence, and verbal intelligence are presented in Table 1. The one-way analysis of variance (ANOVA) on reasoning intelligence across the 3 groups yielded no significant difference: *F*(2, 149) = 0.498, *p* = 0.609, and 95% confidence interval (CI): [ − 1.39, 3.71]. Similarly, no significant difference was observed for verbal intelligence: *F*(2, 149) = 0.981; *p* = 0.378, and 95% CI: [ − 4.34, 3.18], indicating the 3 groups were comparable in terms of reasoning and verbal intelligence.

**TABLE 1:** Descriptive statistics of participants’ age, reasoning intelligence, and verbal intelligence.

Group	Age (year)			Reasoning intelligence	Verbal intelligence
	*n*	Average age	Age range		
Children with ASD	50	10.14 (2.08)	6–15	27.86 (7.67)	81.26 (14.34)
Children with ID	50	10.16 (2.16)	6–15	27.44 (7.36)	79.18 (13.10)
Children with TD	50	4.78 (1.14)	3–6	28.92 (7.10)	79.62 (11.05)

Data are expressed as mean ± (standard deviation). ASD, Autism spectrum disorder; ID, intellectual disability; PPVT, Peabody Picture Vocabulary Test; TD, typical development.

The number of white lie-telling behaviors in real situations for the 3 groups was analyzed, as presented in Table 2.

**TABLE 2:** Descriptive statistics of white lie cognition and empathic ability by group.

Group	White lie cognition		Empathic ability	
	White lie degree evaluation	White lie moral evaluation	Cognitive empathy	Emotional empathy
Children with TD (*n* = 50)	1.34 (0.74)	1.26 (0.77)	3.32 (0.62)	6.70 (0.99)
Children with ASD (*n* = 50)	0.54 (1.05)	-0.01 (1.01)	1.84 (0.65)	3.96 (0.78)
Children with ID (*n* = 50)	0.82 (0.69)	0.34 (1.12)	2.30 (0.67)	4.86 (0.80)

Data are expressed as mean ± (standard deviation). ASD, Autism spectrum disorder; ID, intellectual disability; TD, typical development.

### 3.2 Scores on components of white lie cognition

**FIGURE 3:**
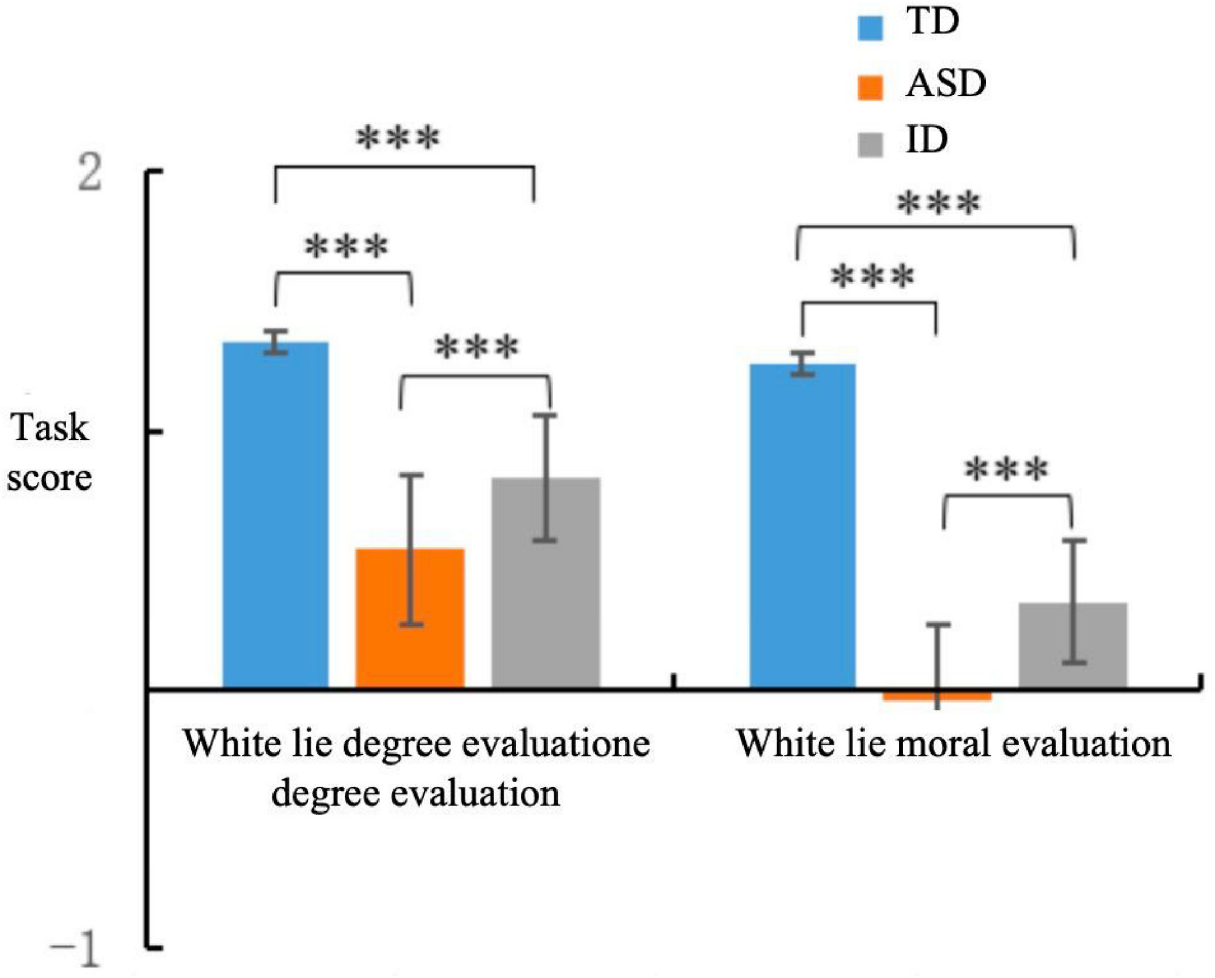
Comparison chart of scores for each component of white lie cognition. ***Denotes highly significant difference, *p* < 0.001.

A one-way repeated measures ANOVA on the components of white lie cognition revealed a significant main effect of the groups (Figure 3), *F*(2, 147) = 19.66, *p* < 0.001, and η^2^ = 0.21. *Post hoc* tests demonstrated that the white lie cognition score was significantly higher for the children with TD group [*M* = 1.30, standard deviation (SD) = 0.12] than for the children with ASD group (*M* = 0.25, *SD* = 0.12), *p* < 0.001, and 95% CI: [0.71, 1.37] and children with ID group (*M* = 0.58, *SD* = 0.11), *p* < 0.001, and 95% CI: [0.38, 0.16]. Furthermore, the white lie cognition score of children with intellectual disability (*M* = 0.58, *SD* = 0.11) was higher than that of children with autism spectrum disorder (*M* = 0.25, *SD* = 0.12), a difference that was marginally significant [*p* = 0.056, 95% CI (-.66, .008)].

A significant main effect of white lie cognition component was also observed, *F*(2, 147) = 52.77, *p* < 0.001, and η^2^ = 0.26. *Post hoc* tests indicated that across all 3 groups, scores for white lie degree evaluation (*M* = 0.90, *SD* = 0.07) were significantly higher than those for white lie moral evaluation (*M* = 0.52, *SD* = 0.08), *p* < 0.001, and 95% CI: [0.27, 0.48].

### 3.3 Empathic ability

**FIGURE 4:**
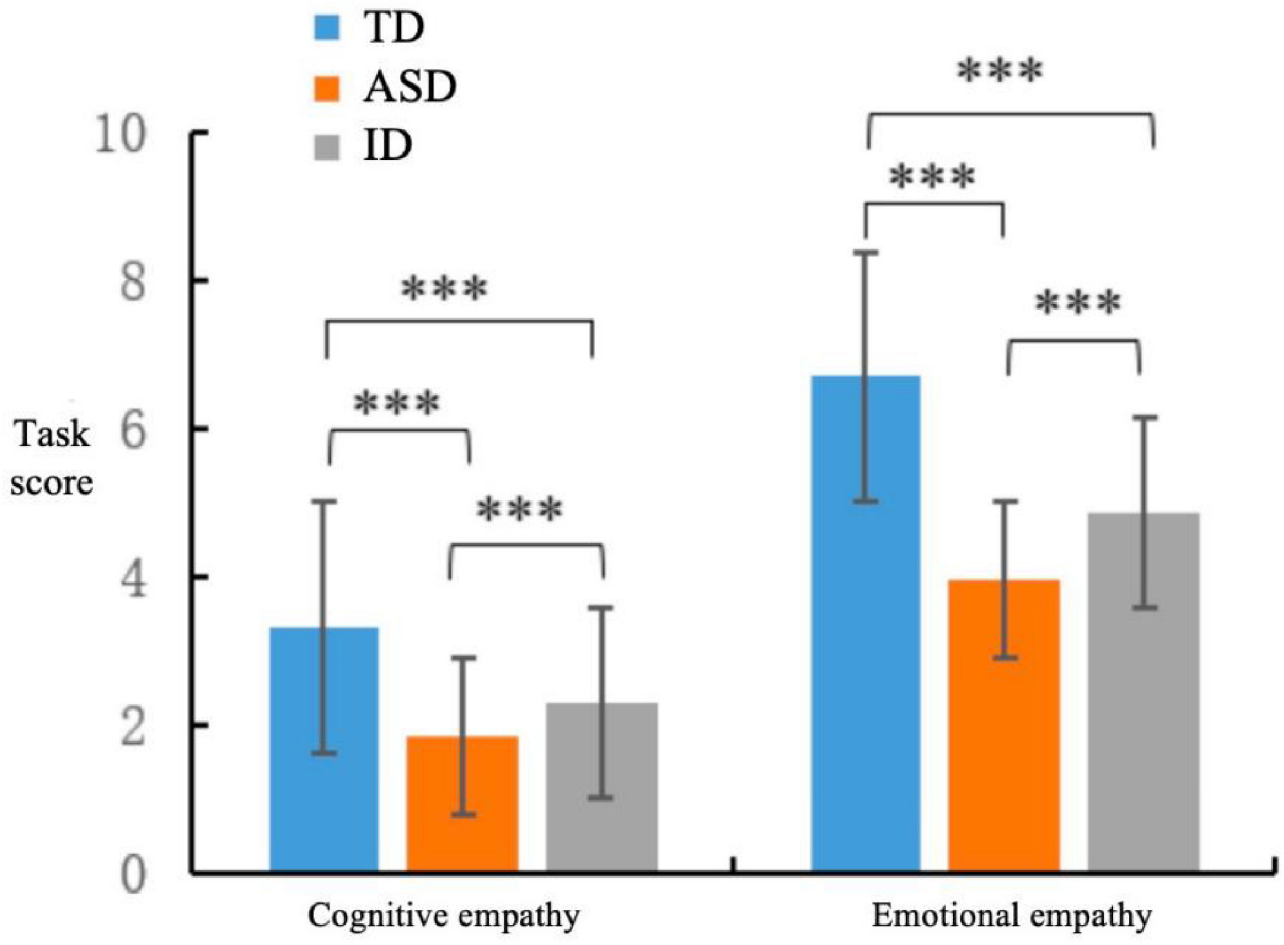
Comparison chart of scores for each component of empathy ability. ***Denotes highly significant difference, *p* < 0.001.

A one-way analysis of variance ANOVA on empathic ability revealed a significant main effect of the groups (Figure 4), *F*(2, 147) = 125.51, *p* < 0.001, and η^2^ = 0.631. *Post hoc* tests demonstrated that the empathic ability score was significantly higher for the children with TD group (*M* = 10.02, *SD* = 1.44) than for the children with ASD group (*M* = 5.80, *SD* = 1.33), *p* < 0.001, and 95% CI: [3.68, 4.76] and children with ID group (*M* = 7.16, *SD* = 1.32), *p* < 0.001, and 95% CI: [2.32, 3.40]. Additionally, the empathic ability score was significantly higher for the children with ID group than for the children with ASD group, *p* < 0.001 and 95% CI: [0.82, 1.90].

A repeated measures ANOVA was conducted with empathic ability as the within-subjects factor and group as the between-subjects factor. The results revealed a significant main effect of empathic component, *F*(1, 147) = 2168.99, *p* < 0.001, η^2^ = 0.937, with scores for emotional empathy (*M* = 5.17, *SD* = 1.43) being significantly higher than those for cognitive empathy (*M* = 2.49, *SD* = 0.90). The interaction effect between empathic component and group was also significant, *F*(2, 147) = 40.96, *p* < 0.001, η^2^ = 0.358, indicating that the discrepancy between cognitive and emotional empathy varied across the three groups.

### 3.4 Relationships between empathic ability and white lie cognition

Multiple hierarchical regression analyses were conducted with scores on the white lie cognition components as dependent variables to examine the influence of empathic ability on white lie cognition. In Step 1, age was entered into the regression equation. In Step 2, cognitive empathy and emotional empathy scores were entered. In Step 3, their interaction term was entered. The results are presented in Table 3.

**TABLE 3:** Multiple linear regression analysis of empathic ability and components of white lie cognition by group.

Group		Variable	White lie degree evaluation					White lie moral evaluation				
			*R* ^2^	Δ*R*^2^	*F*	β	*t*	*R* ^2^	Δ*R*^2^	*F*	β	*T*
TD	Step 1	Age	0.31	0.29	21.24***	0.42	4.61***	0.50	0.49	48.42***	0.57	6.96***
	Step 2	Cognitive empathy				0.77	5.81***				1.05	10.68***
		Emotional empathy				0.49	6.14***				0.57	7.53***
	Step 3	Cog. × Emot. empathy	0.55	0.53	28.55***	0.47	3.36**	0.81	0.80	99.87***	0.78	8.13***
ASD	Step 1	Age	0.16	0.14	8.86**	0.26	2.98**	0.16	0.14	9.16**	0.26	3.03**
	Step 2	Cognitive empathy				1.37	10.87***				1.34	11.71***
		Emotional empathy				1.11	9.92***				1.13	12.69***
	Step 3	Cog. × Emot. empathy	0.81	0.80	100.01***	0.60	4.93***	0.88	0.88	179.16***	0.69	7.62***
ID	Step 1	Age	0.40	0.39	32.01***	0.22	5.66***	0.38	0.37	29.45***	0.35	5.43***
	Step 2	Cognitive empathy				0.65	5.78***				1.11	6.27***
		Emotional empathy				0.71	10.35***				0.82	5.14***
	Step 3	Cog. × Emot. empathy	0.74	0.72	65.08***	0.59	7.58***	0.52	0.50	25.45*	0.44	2.61*

*p < 0.05,

**p < 0.01,

***p < 0.001.

As depicted in Table 3, cognitive empathy, emotional empathy, and their interaction significantly predicted scores on both white lie degree evaluation and white lie moral evaluation for all 3 groups.

## 4 Discussion

### 4.1 High empathic ability facilitates white lie degree evaluation in children with ASD

The results indicated that children with ASD performed more accurately in white lie degree evaluations under conditions of higher empathic ability. This finding suggests that their evaluation of white lie severity is related to empathic ability, aligning with previous research on children with TD (Zhen and Liming, 2024). Furthermore, this study was novel in exploring the impact of empathic ability on white lie cognition in children with ASD, revealing a significant correlation with white lie moral evaluation as well. The findings suggest that empathic ability may represent an alternative cognitive mechanism underlying white lie cognition in this population. Studies on children with TD have also reported a close link between white lie degree evaluation and empathic ability (Lim et al., 2020; Guo and Wu, 2021), with a few scholars positing empathic ability as a necessary cognitive foundation for such evaluations (Decety et al., 2018). This implies that children with ASD who possess stronger empathic ability are more likely to understand the liar’s motivation and recognize the positive emotional outcomes that a well-intentioned lie can produce for the listener. Therefore, enhancing the ability of children with ASD to perceive emotional cues from their environment may help them understand the benevolence embedded in white lie behaviors. The relevance of this finding extends beyond theoretical contributions to practical implications. Although the underlying neural mechanisms were not examined in this study, the findings provide a new direction for future research. Investigating the mechanisms and interventions related to white lie cognition in both children with ASD and TD may help improve individuals’ ability to evaluate white lies, reduce the potential negative impact of “difficulties in recognizing white lies” on social development, and hold crucial theoretical and practical value for both individuals and society. It is worth noting that the predictive role of empathic ability in evaluating the degree of white lies may be closely related to children’s capacity to represent others’ mental states. Previous research has indicated that understanding white lies requires the simultaneous engagement of cognitive empathy (understanding others’ emotions) and theory of mind (understanding others’ beliefs) (Dvash and Shamay-Tsoory, 2014). Future studies could further explore the interaction between empathy and theory of mind in the white lie cognition of children with special needs. In summary, the first key finding of this study was that the white lie degree evaluation in children with ASD was influenced by their own empathic ability. As this area remains underexplored, the stability and validity of this result require further verification and refinement by future research. It is also recommended that future studies use story content closely aligned with the daily experiences of children with ASD to facilitate perspective-taking and moral judgment.

### 4.2 High empathic ability improves white lie moral evaluation in children with ASD

Regression analysis and simple slope tests in this study revealed a marked correlation between white lie moral evaluation and empathic ability in children with ASD. Compared with the low-empathy group, the high-empathy group demonstrated stronger predictive power for moral evaluation, indicating that empathic ability plays a crucial role in this process. Previous studies have reported that children with ASD (even high-functioning autism) perform poorly on white lie cognition tasks, and their parents often report an absence of lying behavior (Sodian and Frith, 1992). However, acquiring an understanding of white lie cognition may facilitate the development of social interaction skills and help establish more harmonious interpersonal relationships in children with ASD (Williams White et al., 2007). This study offered a novel contribution by identifying a significant correlation between white lie moral evaluation and empathic ability in children with ASD. One possible explanation is that both empathic ability and moral evaluation rely on observing and reasoning about others’ behaviors (Lombardo et al., 2007). Although children with ASD can be trained to succeed on basic empathy tasks, they may still perform poorly on more advanced emotion recognition tasks (Christina et al., 2012) and struggle to apply these skills flexibly in daily life (Monica et al., 2014). Additionally, empathic ability typically develops from cognitive to emotional empathy, a process that is often delayed in children with ASD compared with their peers with TD (Schwenck et al., 2012). As a result, their age for competent white lie moral evaluation is also delayed (Lewis, 2015). Therefore, educators should develop a comprehensive understanding of white lie moral evaluation in children with ASD and implement appropriate educational strategies. In addition to teaching appropriate evaluative responses, daily instruction should also explain the reasons and moral factors behind such evaluations, helping them to connect behavior with empathy. In summary, the second key finding of this study was that children with ASD who possessed higher empathic ability were able to engage in white lie moral evaluation.

### 4.3 High empathic ability improves white lie degree evaluation in children with ID

The performance of children with ID demonstrated improved performance in white lie degree evaluation with higher empathic ability, rendering them more likely to form correct judgments. This indicates that children with ID who have stronger empathic ability are more capable of understanding the protagonist’s mental state in a story, thereby comprehending the white lie. Similarly, when assessing the protagonist’s emotional state following a false response, children in the low-empathy group scored significantly lower than those in the high-empathy group. However, because white lies belong to the domain of prosocial behavior, the ability to correctly judge others’ emotional states is a prerequisite for white lie cognition (Schutte et al., 2001). Empathy serves as a crucial motivational basis for white lie behavior, and cultivating empathic ability can facilitate a more accurate degree evaluation (Cameron et al., 2022). Compared with understanding other’s beliefs, recognizing and interpreting the protagonist’s emotions in a story might be more crucial for white lie cognition (Hsu and Cheung, 2013). In educational settings, teachers can appropriately enhance the understanding of white lies among children with ID by using the cultivation of empathic ability as a starting point to promote the development of their white lie degree evaluation. When children with ID engage in white lie behaviors, educators should acknowledge their positive intent and provide timely, positive guidance and encouragement. In summary, the third key finding was that the empathic ability of children with ID positively predicted their white lie cognition, particularly the degree evaluation under conditions of high empathic ability.

### 4.4 High empathic ability improves white lie moral evaluation in children with ID

A major innovation of this study is the inclusion of empathic ability to investigate white lie cognition in children with ID. Several previous studies have demonstrated that improving empathic ability facilitates the enhancement of white lie cognition in children with TD (Zhen and Liming, 2024). Building on this foundation and expanding the participant pool, this study selected children with ID as subjects to compare differences among children with ID, ASD, and TD in this aspect. The study also observed that for children with TD, higher empathic ability scores were associated with higher white lie moral evaluation scores. Similarly, for children with ID, higher empathic ability scores correlated with higher moral evaluation scores. This result suggests that possessing higher empathic ability will aid children with ID in performing white lie moral evaluation (Talwar and Crossman, 2011). Taken together, this study is the first to directly compare the white lie cognition mechanisms of children with autism spectrum disorder (ASD) and children with intellectual disability (ID) within the same experimental paradigm. The results revealed that although the two groups were matched on basic cognitive abilities, children with ASD scored lower than children with ID on both empathy and white lie cognition—a pattern that may be related to the unique core social cognitive deficits associated with ASD. More importantly, the predictive patterns of empathic ability on white lie cognition were consistent across the two groups, suggesting that empathy serves as a shared cognitive foundation for understanding white lies in both clinical populations. This finding not only extends previous research conclusions—largely based on children with typical development—to special populations, but also provides a more precise basis for targeted social cognitive interventions. In summary, the fourth key finding was that children with ID who exhibited stronger empathic ability were capable of accurate white lie moral evaluation. This clearly indicates that when studying the development of ID with intellectual disabilities’ understanding of white lies, the factor of empathy should be taken into consideration. This result contributes to a deeper understanding of the mechanism underlying white lie cognition in this population.

### 4.5 Limitations and educational implications

This study had several limitations that should be addressed in future research. The present study has several limitations that should be addressed in future research. First, although the three groups differed in chronological age, we matched participants on reasoning intelligence and verbal intelligence using the Raven’s Progressive Matrices and the Peabody Picture Vocabulary Test. This ensured that there were no significant differences in basic cognitive levels among the three groups, thereby providing a relatively comparable cognitive starting point for cross-group comparisons of white lie cognition and empathic ability. However, age remains an important indicator of sociocognitive development. Future studies could further employ mental age matching or developmental trajectory tracking designs to more precisely reveal the uniqueness and developmental delays in the white lie cognition of children with special needs. Third, this study only investigated the degree evaluation and moral evaluation components of white lie cognition, without assessing the development of white lie behavior. Future research should examine the relationship between these 2 subcomponents of cognition and white lie behavior in children with ASD and ID. Finally, the exploration of the relationship between empathic ability and white lie cognition in this study was limited to experimental methods, without addressing the underlying neural mechanisms. Future research should employ neuroimaging techniques such as functional MRI to investigate these neural mechanisms.

Despite these limitations, the findings of this study have crucial educational implications. First, teachers can incorporate more role-playing games in the classroom (Volbrecht et al., 2007); acting out a role helps improve understanding of that role. Parents can likewise encourage children with ASD and ID to consider and imagine others’ feelings and experiences during social interactions (Ozdemir, 2008). At the same time, greater emphasis should be placed on cultivating empathic ability in children with ASD and ID. This cultivates healthy social emotions and can, to a certain extent, improve emotion regulation ability and inhibit the occurrence of deviant behaviors (Bagnall et al., 2024). Second, educators need to correctly view the development of white lie cognition, clarify its role in the social development of children with ASD and ID, seek educational opportunities in daily life to foster a correct understanding of white lies, and help them learn to use white lie strategies appropriately and timely (Kokina and Kern, 2010). Finally, technologies such as Virtual Reality, computers, and social robots offer promising tools for enhancing empathic ability and white lie cognition in children with ASD and ID (Cunha et al., 2018). In particular, social interactive robots can provide structured training in empathic ability and white lie cognition while also serving as social partners for effective communication, thereby promoting the development of white lie cognition through interactive skills. Finally, as this study employed self-reported data, common method bias was a potential concern. Procedural remedies such as ensuring respondent anonymity and incorporating reverse-scored items were implemented during data collection to mitigate this bias. A statistical test was performed using Harman’s single-factor test. The factor analysis extracted 4 factors with eigenvalues > 1. The largest factor accounted for 35.11%, < the 40% threshold, indicating no serious common method bias in the data.

## 5 Conclusion

(1) Compared with children with typical development, children with autism spectrum disorder and children with intellectual disability performed less well on both white lie degree evaluation and white lie moral evaluation.

(2) Empathic ability positively predicted white lie cognition in children with autism spectrum disorder and children with intellectual disability, with emotional empathy making a particularly prominent contribution. Children with higher empathic ability demonstrated more positive performance in both white lie degree evaluation and moral evaluation.

## Data Availability

The original contributions presented in this study are included in the article/supplementary material, further inquiries can be directed to the corresponding authors.
